# The Early Innate Response of Chickens to *Salmonella enterica* Is Dependent on the Presence of O-Antigen but Not on Serovar Classification

**DOI:** 10.1371/journal.pone.0096116

**Published:** 2014-04-24

**Authors:** Karolina Varmuzova, Marta Elsheimer Matulova, Alena Sebkova, Zuzana Sekelova, Hana Havlickova, Frantisek Sisak, Vladimir Babak, Ivan Rychlik

**Affiliations:** Veterinary Research Institute, Brno, Czech Republic; University of Osnabrueck, Germany

## Abstract

*Salmonella* vaccines used in poultry in the EU are based on attenuated strains of either *Salmonella* serovar Enteritidis or Typhimurium which results in a decrease in *S*. Enteritidis and *S*. Typhimurium but may allow other *Salmonella* serovars to fill an empty ecological niche. In this study we were therefore interested in the early interactions of chicken immune system with *S*. Infantis compared to *S*. Enteritidis and *S*. Typhimurium, and a role of O-antigen in these interactions. To reach this aim, we orally infected newly hatched chickens with 7 wild type strains of *Salmonella* serovars Enteritidis, Typhimurium and Infantis as well as with their *rfaL* mutants and characterized the early *Salmonella*-chicken interactions. Inflammation was characterized in the cecum 4 days post-infection by measuring expression of 43 different genes. All wild type strains stimulated a greater inflammatory response than any of the *rfaL* mutants. However, there were large differences in chicken responses to different wild type strains not reflecting their serovar classification. The initial interaction between newly-hatched chickens and *Salmonella* was found to be dependent on the presence of O-antigen but not on its structure, i.e. not on serovar classification. In addition, we observed that the expression of calbindin or aquaporin 8 in the cecum did not change if inflammatory gene expression remained within a 10 fold fluctuation, indicating the buffering capacity of the cecum, preserving normal gut functions even in the presence of minor inflammatory stimuli.

## Introduction

The prevalence of *Salmonella enterica* serovar Enteritidis (*S*. Enteritidis) in poultry flocks is gradually decreasing in EU member states [1]. One of the reasons is the use of vaccination in egg-producing flocks, usually with live, attenuated *Salmonella* vaccines based on attenuated strains of *S*. Enteritidis. However, there are concerns that the decrease in *S*. Enteritidis due to successful vaccination may allow other *Salmonella* serovars to fill an empty ecological niche in poultry flocks. One such serovar is serovar Infantis. Isolates of this serovar can be isolated both from pigs and poultry, and are relatively common also in humans [2], [3]. However, as isolates of serovar Infantis are recovered from outbreaks at a lower frequency when compared with *S*. Enteritidis, *S*. Infantis is generally considered as less virulent for the above mentioned host species.

The response of chickens to oral infection with *Salmonella* is characterized by a moderate inflammatory response in the cecum. This response is accompanied by leukocyte infiltration and an increase in the expression of proinflammatory cytokines or immune effectors such as inducible NO synthase [4]–[6]. The response in the chicken cecum, however, is not limited to cytokine gene expression and, in agreement with this, we recently described over 40 new chicken genes that respond highly to *S*. Enteritidis infection [7], [8]. Although the biological function of most of these genes has not been experimentally proven in chickens, analyzing their expression profiles in an integrated fashion may represent a sensitive tool for the characterization of a range of inflammatory signals in the chicken cecum after *Salmonella* infection. Such an approach has been used in this study for characterizing in detail the interactions of chickens with altogether 7 strains belonging to *Salmonella* serovars Enteritidis, Typhimurium and Infantis. In addition, as O-antigen is an obvious difference among *Salmonella* serovars and since LPS and O-antigen influence strain virulence by affecting invasiveness in eukaryotic cells and protein secretion [9]–[11], *rfaL* (*waaL*) mutants disabled in O-antigen synthesis were constructed. Since these mutants were constructed in *S*. Enteritidis (O9 antigen), *S*. Typhimurium (O4 and O5 antigens) and *S*. Infantis (O6 and O7 antigens), we could compare the influence of different O-antigen structures on chicken recognition of *Salmonella*. Using such an approach, we found that all wild type strains stimulated a greater inflammatory response than any of the *rfaL* mutants. However, there were great differences in chicken responses to different wild type strains independent of their serovar classification. The early interaction between chickens and *Salmonella* was therefore dependent on the presence of O-antigen but not on its structure.

## Results

### Bacterial Strains, qRT-PCR

**Figure 1:**
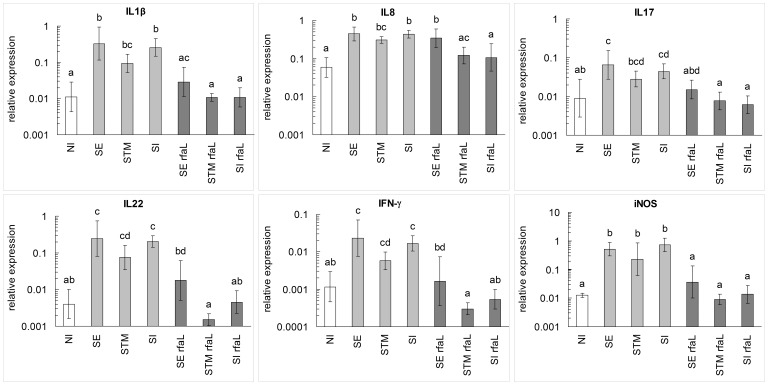
Cytokine gene expression in the cecum of orally infected chickens. Columns represent geometric means of the relative expressions of respective genes. Vertical bars represent 95% confidence intervals regarding the geometric means. Superscripts above columns denote statistically significant differences among groups (columns sharing the same superscript are not significantly different from each other, columns that have no superscript in common are significantly different from each other). NI, expression in the non-infected chickens. SE, expression in the chickens infected with *S*. Enteritidis 147. STM, expression in the chickens infected with *S*. Typhimurium LT2. SI, expression in the chickens infected with *S*. Infantis 1516. SE *rfaL*, expression in the chickens infected with *S*. Enteritidis *rfaL* mutant. STM *rfaL*, expression in the chickens infected with *S*. Typhimurium *rfaL* mutant. SI *rfaL*, expression in the chickens infected with *S*. Infantis *rfaL* (I) mutant. Mind logarithmic scaling of Y-axis.

Initially the chicken response to *Salmonella* serovars Enteritidis, Typhimurium and Infantis and *rfaL* mutants was characterized by real time PCR quantification of IL1β, IL8, IL17, IL22, IFN-γ and iNOS expression in the cecum. Gene expression in the cecum of chickens infected with wild type *Salmonella* strains significantly increased in comparison with that in the non-infected control chickens. *S*. Enteritidis and *S*. Infantis induced the same inflammatory response whilst the chicken response to infection with *S*. Typhimurium LT2 was lower in comparison to the response to other wild type strains. Unlike the wild type strains and except for IL8, the *rfaL* mutants induced a significantly lower level of inflammation which was nearly the same as in the non-infected control chickens (Fig. 1).

### SDS-PAGE of Secreted Proteins

**Figure 2:**
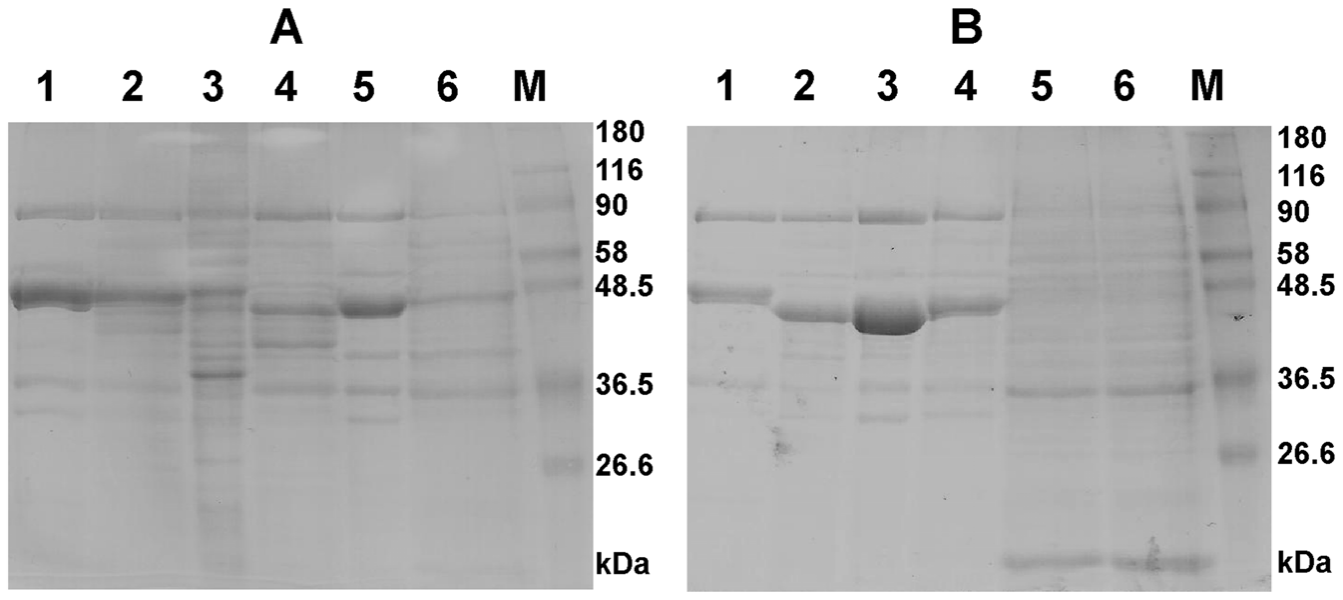
*Salmonella* secreted proteins. Panel A, strains included in the initial part of this study. 1, *S*. Enteritidis 147. 2, *S*. Enteritidis 147 *rfaL* mutant. 3, *S*. Typhimurium LT2. 4, *S*. Typhimurium LT2 *rfaL* mutant. 5, *S*. Infantis 1516. 6, *S*. Infantis 1516 *rfaL* (I) mutant. Lane M, molecular weight standard. This analysis was repeated 3 times for each strain or mutant with similar results in each of the replicates. Panel B, strains included in the second part of this study. 1, *S*. Enteritidis G1481. 2, *S*. Typhimurium 2002. 3, *S*. Typhimurium 2454. 4, *S*. Infantis 514. 5, *S*. Infantis 1516 *rfaL* (II) mutant. 6, *S*. Infantis 1516 *rfaL* (III) mutant. Lane M, molecular weight standard. This analysis was repeated 3 times for each strain or mutant with similar results in each of the replicates.

Since *S*. Typhimurium LT2 induced a lower inflammatory response and the inflammation is dependent on the SPI1 encoded type III secretion system [7], [12], we verified *in vitro* protein secretion for all the strains. Secreted proteins of *S*. Enteritidis 147 and *S*. Infantis 1516, as well as *rfaL* mutants of *S*. Enteritidis 147 and *S*. Typhimurium LT2 were similar to those of other *Salmonella* strains [10], [13], though protein secretion was slightly less efficient in the *rfaL* mutants. On the other hand, the protein profiles of both *S*. Typhimurium LT2 and *rfaL* mutant of *S*. Infantis 1516 were characteristic by a high protein background indicating lysis of the bacterial cells (Fig. 2A). Because of this, in the next set of experiments, we i) extended the number of wild type strains, ii) constructed two additional *rfaL* mutants in *S*. Infantis 1516, iii) verified protein secretion *in vitro* and iv) finally infected chickens with the extended set of mutants and characterized their immune response by monitoring of gene expression of 43 different genes.

Control SDS-PAGE showed that all newly included wild type strains, namely *S*. Enteritidis G1481, *S*. Typhimurium 2002, *S*. Typhimurium 2454 and *S*. Infantis 514, exhibited standard profiles of *Salmonella* secreted proteins. However, two newly constructed *rfaL* mutants in *S*. Infantis 1516 again exhibited a high protein background indicating that this was a common characteristic of *rfaL* mutants of *S*. Infantis 1516 (Fig. 2B).

### Principal Component Analysis (PCA) of Individual Chickens based on their Gene Expression in the Cecum

**Figure 3:**
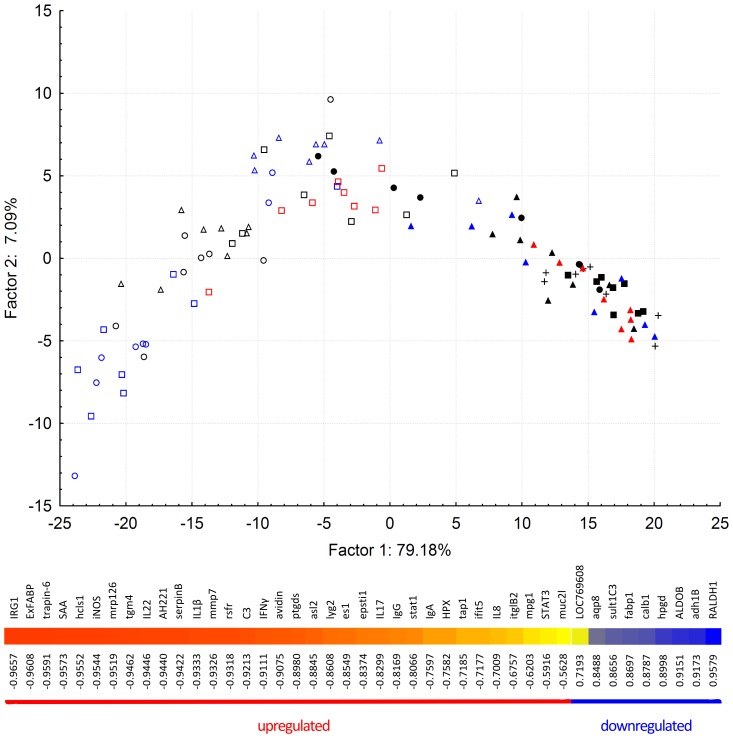
PCA plot of the chickens clustered according to their gene expression in the cecum and heat map correlation coefficients between factor 1 and individual gene expression. Open black circles, *S*. Enteritidis 147; Open blue circles, *S.* Enteritidis G1481; open black squares, *S*. Typhimurium LT2; open blue squares, *S*. Typhimurium 2002; open red squares, *S*. Typhimurium 2454; open black triangles, *S*. Infantis 1516; open blue triangles, *S*. Infantis 514; closed black circles, *S*. Enteritidis 147 *rfaL* mutant; closed black squares, *S*. Typhimurium LT2 *rfaL* mutant; closed black triangles, *S*. Infantis 1516 *rfaL* (I) mutant; closed blue triangles, *S*. Infantis 1516 *rfaL* (II) mutant; closed red triangles, *S*. Infantis 1516 *rfaL* (III) mutant. symbol “plus”, non-infected chickens. PCA also showed that a single factor explained nearly 80% of the variation in the chicken response. This factor was the scope of inflammation itself as high and significant correlations were observed between the expression of individual genes and the positioning of corresponding chickens along X axis. Genes are arranged from the most positively correlated to the most negatively correlated ones.

In the next step, the gene expression of 43 genes was characterized in the caeca of 8 non-infected controls and in the 96 chickens which were infected with all 7 wild type strains and 5 *rfaL* mutants. This dataset was analyzed by PCA (for individual gene expression profiles see Table S1) which showed that all non-infected chickens clustered separately from those infected with any of the wild *Salmonella* strains. Inactivation of *rfaL* attenuated mutants to such an extent that chickens infected with them overlapped with the non-infected controls. The only exception was the *rfaL* mutant of *S*. Enteritidis which stimulated an inflammatory response similar to the least virulent wild type strains. Rather surprisingly, we did not observe any clustering of chickens based on infection with strains belonging to different serovars (Fig. 3).

### Correlation between Salmonella Counts and Gene Expression

**Figure 4:**
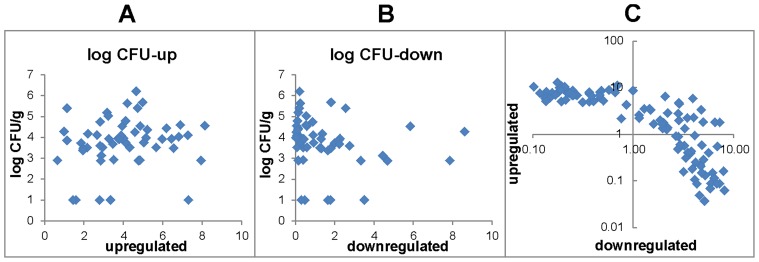
Correlation between gene expression and *Salmonella* counts in the liver (log CFU/g), and correlation between upregulated and downregulated genes. Each dot represents a single chicken characterized by *Salmonella* counts in the liver, average expression calculated from expression of all genes which positively respond to *Salmonella* infection or average expression calculated from expression of all genes which negatively respond to *Salmonella* infection. A, correlation between average expression of upregulated genes and *Salmonella* counts in the liver. B, correlation between average expression of downregulated genes and *Salmonella* counts in the liver. C, correlation between average expression of upregulated and downregulated genes.

Finally we were interested to what extent the inflammatory response might be influenced by total *Salmonella* counts present in infected chickens. To address this question we calculated an average expression of all upregulated or downregulated genes (for all up- or downregulated genes see Fig. 3 or Table S2) and used this value as an index which was plotted against *Salmonella* counts in the liver. *Salmonella* counts in the liver, instead of the cecum, were used as the cecal samples occasionally exhibited overgrowth by other microbiota even on XLD agar supplemented with nalidixic acid. Secondly, *rfaL* mutants were excluded from this analysis as these did not grow on XLD agar. This analysis showed that inflammation induced by the wild type strains was not dependent on *Salmonella* counts in the liver as chickens exhibiting similar *Salmonella* counts in liver responded by a variable expression of marker genes in the cecum (Fig. 4A and 4B).

In the last analysis we plotted the average expression values of upregulated genes against the average expression values of downregulated genes after *Salmonella* infection. This analysis showed that within one log in which the inducible genes changed in expression, the expression of suppressible genes remained unchanged. Only when the expression of inducible genes increased more than 10 fold, the expression of the suppressible genes began to decreases correspondingly. As the downregulated genes are associated with basal gut function such as nutrient transport in the cecum, this means that these functions remain unaffected within minor changes in inflammatory status and only if this extends over a one log fluctuation, normal gut functions become affected (Fig. 4C).

## Discussion

In the present study, the immune response of the gut newly hatched chickens to infection with 7 strains of 3 different *Salmonella* serovars and their O-antigen defective mutants was compared. Unlike the previous reports concluding there was a decreasing invasion and/or inflammatory response of chickens or chicken cell lines to the infection with *S*. Enteritidis, *S*. Typhimurium and *S*. Infantis [4], [6], [14], [15], we did not observe such a serovar-dependent decrease. Instead, recognition of *Salmonella* by newly hatched chickens was more dependent on individual strains and even the pigeon, i.e. bird-adapted, *S*. Typhimurium isolate of phage type DT2 [16] stimulated a lower inflammatory response than the human *S*. Typhimurium isolate of phage type DT104. Some of these interactions might be explained by the known lower virulence of some strains such as *S*. Typhimurium LT2 due to a mutation in the *rpoS* start codon [17], [18], lower stability in the stationary phase [19] and release of cytoplasmic proteins into the medium (Fig. 2). However, if taken collectively, the early interaction between naïve chickens and *Salmonella* was not affected by serovar but rather by particular strain characteristics. This, however, does not exclude that there is a systemic serovar-dependent chicken response in sites such as the liver and spleen, or in the cecum but at later stages of infection.

Removal of O-antigen significantly reduced the ability of *Salmonella* to induce inflammation in the chicken gut, likely due to their increased sensitivity to antimicrobial peptides or serum [20]. The *rfaL* mutant of *S*. Enteritidis 147 exhibited the highest residual virulence whilst the removal of O-antigen from *S*. Typhimurium LT2 and *S*. Infantis 1516 nearly abolished the ability of these mutants to induce inflammation in infected chickens as these clustered closely to the non-infected controls. It would be interesting to test whether the low level inflammation induced by the *rfaL* mutants, that of *S*. Enteritidis in particular, would be able to protect chickens against systemic site colonization after subsequent challenge by wild type strains administered 24 hours later, as observed in gnotobiotic piglets [21].

Finally we analyzed whether the different inflammatory responses could be affected by *Salmonella* counts in liver. For such analysis we separately analyzed the genes which were induced or suppressed in the cecum of *Salmonella* infected chickens [7]. The former are associated with the innate immune response and inflammation and the latter are associated with normal gut function such as transport of calcium or water (e.g. calbindin1 and aquaporin 8). However, as plotting the *Salmonella* counts either against an average expression of all upregulated genes, or against an average expression of all downregulated genes did not show any clear profile, a simple mechanistic explanation based on *Salmonella* counts is not appropriate.

Interestingly, when the average expression level of *Salmonella* upregulated genes was plotted against the average expression level of *Salmonella* downregulated genes in individual chickens, we noticed that the expression of suppressible genes did not change in expression as long as the expression of the inducible genes increased more than tenfold. Only when the inducible gene expression increased more than tenfold, the expression of suppressible genes began to decline, too. Since the suppressible genes represent normal functions of the chicken cecum, this shows that there is a “buffering” capacity in the chicken cecum preserving its normal function even in the presence of minor inflammatory fluctuations.

## Materials and Methods

### Ethical Statement

The handling of animals in the study was performed in accordance with current Czech legislation (Animal protection and welfare Act No. 246/1992 Coll. of the Government of the Czech Republic). The specific experiments were approved by the Ethics Committee of the Veterinary Research Institute (permit number 48/2010) followed by the Committee for Animal Welfare of the Ministry of Agriculture of the Czech Republic (permit number MZe 1226).

### Bacterial Strains

All bacterial strains used in this experiment were spontaneously resistant to nalidixic acid and are listed in Table 1. *rfaL*::Cm mutations were constructed by λ red recombination [22] and verified by PCR using primers designed to amplify through the inserted gene cassette – inactivated gene junction.

**Table 1:** List of *Salmonella enterica* strains and their *rfaL* mutants used in this study.

Serovar	Strain ID	Mutant	O antigen	Phage type
Enteritidis 147	7F4	wt	1,9,12	PT4
Enteritidis G1481	2I2	wt	1,9,12	PT4
Typhimurium LT2	11F4	wt	1,4,5,12	ND*
Typhimurium 2002	16E5	wt	1,4,5,12	DT104
Typhimurium 2454	15B4	wt	1,4,5,12	DT2
Infantis 1516	18G6	wt	6,7,14	ND
Infantis 514	18F10	wt	6,7,14	ND
Enteritidis 147	14E5	Δ*rfaL*::Cm	none	ND
Typhimurium LT2	7F10	Δ*rfaL*::Cm	none	ND
Infantis 1516 (I)	21A2	Δ*rfaL*::Cm	none	ND
Infantis 1516 (II)	22C9	Δ*rfaL*::Cm	none	ND
Infantis 1516 (III)	22C10	Δ*rfaL*::Cm	none	ND

*ND, not determined.

### Analysis of Secreted Proteins

Protein secretion was tested by precipitation of *Salmonella* secreted proteins with trichloracetic acid and their resolution by sodium dodecylsulphate-polyacrylamide gel electrophoresis [17]. In brief, bacterial strains were grown in LB broth at 37°C for 16 hours. The bacterial culture (25 ml) was centrifuged at 4 500×g for 15 min at 4°C. The supernatant was filtered through Millex GP 0.22 µm pore sized filter (Millipore) and proteins were precipitated with trichloroacetic acid at a final concentration of 13%. After 60 min, the precipitated proteins were harvested by centrifugation at 4 500×g for 10 minutes at 4°C, washed twice with acetone and electrophoresed by SDS-PAGE using a 12% polyacrylamide gel followed by Coomassie blue staining.

### Experimental Animals

Newly hatched male ISA Brown chickens (Hendrix Genetics, Boxmeer, Netherlands) were used in this study. The chickens were reared in perforated plastic boxes at 22°C with free access to water and pathogen-free feed. Each of the experimental or control groups was kept in a separate room.

Four chickens per group were orally infected on day one of life with 10^7^ CFU of *Salmonella* strains listed in Tab. 1. In addition, four non-infected chickens were included as a negative control group. Four days later, the chickens were sacrificed and approximately 25 mg of cecal wall was collected into RNALater solution (Qiagen) and stored at −80°C. In parallel, 0.5 g of liver tissue or cecal content was processed for *Salmonella* enumeration. These samples were homogenized in peptone water, tenfold serially diluted and plated on XLD agar plates (HiMedia) supplemented with 20 µg/ml nalidixic acid. Samples negative after direct plating were subjected to a pre-enrichment in buffered peptone water and enrichment in modified semi-solid Rappaport-Vassiliadis medium (Oxoid) for qualitative *Salmonella* strains and mutant determination. *Salmonella* counts positive after direct plating were logarithmically transformed. Samples positive only after enrichment were assigned a value of 1 and negative samples were assigned a value of 0. The chicken infections were performed twice so that we obtained data for the chicken response for each strain or mutant on two completely independent occasions. The non-infected control chickens were also processed for *Salmonella* enumeration always yielding negative results.

### RNA Purification and Quantitative Real-rime PCR

Approx. 25 mg of cecal wall was transferred into 1 ml of TRI reagent (Molecular Research Centre) and homogenized using zirconia silica beads (BioSpec Products) in a MagNALyser (Roche). Fifty µl of bromanisole (Molecular Research Centre) was added to the homogenate, the samples were vigorously shaken for 10 s and centrifuged at 4°C for 15 min at 12 000×g. The upper aqueous phase (500 µl) was collected and mixed with an equal volume of 70% ethanol. This mixture was added to RNeasy purification columns (Qiagen) and washing and RNA elution was performed exactly as recommended by the manufacturer. The concentration and purity of purified RNA was determined spectrophotometrically (Nanodrop, Thermo Fisher Scientific). One µg of RNA was immediately reverse transcribed into cDNA using M-MLV reverse transcriptase (Invitrogen) and oligo (dT) primers. After reverse transcription, the cDNA was diluted 10 times with sterile water and stored at −20°C until real-time PCR. Real-time PCR was performed in 3 µl volumes on 384-well microplates using QuantiTect SYBR Green PCR Master Mix (Qiagen) and a Nanodrop pipetting station from Inovadyne for PCR mix dispensing. The amplification of PCR products and signal detection were performed using a LightCycler II (Roche) with an initial denaturation at 95°C for 15 min followed by 40 cycles of 95°C for 20 s, 60°C for 30 s and 72°C for 30 s. Each sample was subjected to real-time PCR in duplicate and the mean values of the duplicates were used for subsequent analysis. The Ct values of the genes of interest were normalized (ΔCt) to an average Ct value of three house-keeping genes, GAPDH (glyceraldehyde-3-phosphate dehydrogenase), UB (ubiquitin) and TBP (TATA box-binding protein) [23], [24], and the relative expression of each gene of interest was calculated as 2^−ΔCt^. These expression levels were used for data analysis. The initial characterization of the immune response to infection with *S*. Enteritidis 147, *S*. Typhimurium LT2, *S*. Infantis 1516 and their *rfaL* mutants was based on the quantification of IL1β, IL17, IL22, IL8, IFNγ and iNOS transcripts. In the second part of this study, the expression of 43 different genes was used for complex characterization of the chicken immune response to infection with all the *Salmonella* strains. All the primer sequences are listed in Table S2.

### Reproducibility and Statistics

ANOVA followed by post-hoc Tukey's HSD test were used for the analysis of data obtained during initial infections with *S*. Enteritidis 147, *S*. Typhimurium LT2 and *S*. Infantis 1516 and their *rfaL* mutants. Principal component analysis (PCA) was used later to characterize the integrated immune response of chickens based on the expression of 43 genes in 8 chickens for each of the 13 groups including the non-infected controls. The PCA was calculated using unscaled ΔCt values, i.e. we used covariances in an association matrix. All the statistical analyses have been performed using Statistica v. 9.1 (StatSoft) software.

## Supporting Information

Table S1 **Complete list of gene expression for each gene and chicken.**
(XLS)

Table S2 **List of primers used in SybrGreen real time reverse transcription PCR in this study.**
(XLS)
